# Recherches Physiologiques et Medicales sur les Causes et les Symptomes, et le Traitement de la Gravelle

**Published:** 1833-01-01

**Authors:** 


### Recherches Physiologiques et Médicales sur les Causes, les Symptomes, et le Traitement de la Gravelle. Par *F. Majendie.* 2d edit. 1828, p. 151.

Of all animal products the urine perhaps has attracted the most attention, not only on account of the facility with which it is subjected to examination, but also from its connexion with diseases, a fact recognized by the earliest medical writers. To the more scientific analysis of this fluid by modern animal chemists must be attributed the beneficial changes which have been produced in the treatment of its diseases; proving the benefits which result from the attentive study of animal chemistry, as well as the necessity of an accurate knowledge of this branch of medical science to the practitioner. The physician is as well acquainted with the composition of gall-stones, as with that of the various urinary concretions; and why should he not be as able to prevent the formation of cholesterine as of uric acid? This is owing in great measure to his ignorance of the chemical changes produced in the bile by disease; his knowledge of that secretion is almost wholly confined to its healthy state, for the analysis is made of the contents of the gall bladder removed after death; in practice he is content with observing the colour alone of the excrement which is tinged by this fluid, and thus his examination is confined to the most unimportant part—the colouring principle. The difficulties which oppose the examination of the pathological condition of bile are probably insurmountable, and unless the animal chemist had the unwearied patience of Swift's philosopher of Laputa, who spent his whole life in endeavouring to extract from fæces their nutritive principles, it is to be feared that our knowledge of bile, will, for at least a long period, be confined to its normal state. The attempts of the older writers to account for the formation of urinary concretions were generally as ridiculous in theory, as they were useless in practice. Hippocrates imagined that the neck of the bladder became inflamed, and refused exit to all but the thinnest part of the retained urine, whilst the thickest subsided forming calculi. Paracelsus believed them to be produced by an animal resin hardened by the spirit of urine! Even Van Helmont, in the 17th century, who was most inclined to explain the phenomena of health and of disease by chemical laws, compared the formation of gravel to the deposition of tartar from urine. Scheele, in 1776, was the first who discovered the true nature of urinary concretions, he proved that they were most frequently formed by a particular acid which he named lithic, and which he recognized as one of the ingredients of healthy urine. Fourcroy and Vauquelin, in France, Wollaston, Marcet, Proust, Brande and Prout in our country, have since confirmed the discoveries of the Swedish chemist, and added much to our knowledge of both the healthy and morbid principles of the urine. M. Majendie has in the work before us applied some of his physiological discoveries to actual practice, and has endeavoured to explain in what manner various foods act in producing depositions in the urine. It has been too much the fashion in this country to attribute almost invariably the various forms of sand or gravel to indigestion: M. Majendie has explained in a much more satisfactory manner the action of animal food, and of all those articles of diet which contain nitrogen; he candidly confesses his inability to explain many causes of gravel which are not comprehended in this class, but he falls into an opposite error by denying that a disordered secretion of urine can be produced by a disordered state of that organ which prepares the pabulum for the kidneys themselves. We shall give a careful analysis of this work, which is the only one which M. Majendie has published as a practical physician; his reputation as a physiologist alone would command attention to a subject so closely related to his chief pursuits. It embraces the causes, symptoms, and treatment of gravel. When a person discharges habitually or at intervals with his urine, a sand, most frequently of a reddish colour, or small stones, of various forms, colour, and size, he is attacked with gravel. It is a disease more particularly of middle and of old age, those who are fond of the pleasures of the table, and who take little exercise are most subject to it. In some cases there is little inconvenience or pain except at the moment of expulsion, when there is slight ardor urinæ, but usually there is general indisposition, often fever, acute pains in the lumbar region and in the course of the ureters, suppression of urine, a copious flow of blood per urethram, want of sleep, disturbance of all the functions; these symptoms last many days, and do not entirely cease until after the expulsion of one or more gravel stones. If such fits of gravel are frequent or constant it is not only a painful disease, but one likely to shorten life. It is but the first stage of many very intractable diseases, as stone in the pelvis of the kidney, or in the bladder, for which lithotomy or lithotrity are mere palliatives as the cause remains. When this disease is prolonged it may produce nephritis, retention of urine, abscess, urinary fistulæ, hematuria, &c.

### ON THE FORMATION OF GRAVEL.

Urine consists of water holding in solution a certain number of different substances which have a greater or less tendency to leave the liquid in which they are dissolved, and to precipitate themselves under various forms. Sand, gravel, small calculi, &c. are substances which the urine had held in solution, and which are precipitated within the urinary passages. Sand and gravel differ in many important particulars. 1. In size: they vary from a small hazel-nut to the finest dust. 2. In form: some gravel stones are polished spheres; others are pyriform, with their surface smooth or rough; others are angular fragments, evidently detached from larger calculi in the bladder, of which they have formed one of the concentric layers, most frequently they are isolated, and are expelled successively; in a few cases they have been connected by hairs. 3. In colour; generally a yellow red; sometimes a yellowish white, or a cindery grey; very rarely a deep brown or almost black. 4. In consistence. In some instances they are extremely hard, in others they crumble beneath the slightest pressure. 5. In opacity or transparency. M. Majendie found in one case transparent gravel, with this exception, it is opaque.

*Chemical Composition of Gravel.* 1. It is most frequently composed of uric acid united to a small quantity of animal matter. 2. Of the salts which are contained in healthy urine; these are the ammoniaco-magnesian phosphate, and phosphate of lime united to a small quantity of phosphate or carbonate of magnesia. 3. Of substances which are foreign to healthy urine; these are oxalate of lime, cystic oxide, fibrine, and hairs. M. Majendie has found six species of gravel. The red, white, grey, yellow, hairy, and transparent.

1. *Red Gravel.* In order to comprehend the origin, and to establish the basis of successful treatment of this species of gravel which is by far the most frequent, it will be necessary to enter into the history of uric acid.

The urine of man, and of many animals which are nourished with highly azotized foods contains uric acid. Its proportion varies according to the nature of the food; in those animals whose diet is wholly animal, the urine is abundantly charged with it, and even is entirely formed of it, as the researches of Vauquelin and Wollaston, on the urine of birds, have shewn. Still no uric acid has been found in the urine of the lion and tiger, but urea in great abundance. In truth, the nature of the food does not influence the secretion of uric acid alone, but also that of the other substances in solution in the urine, a fact which is of the highest importance in ascertaining the causes of gravel. If the food of animals is herbiverous no trace of uric acid is found. In a paper which M. Majendie read before the Academy of Sciences, some years ago, a series of experiments was detailed which shewed that, by depriving a carnivorous animal of all azotized food, and by feeding it on sugar, gum, oil, and similar substances which are considered as nutritious, but contain no nitrogen, the urine at the end of three or four weeks was deprived of uric acid. A small, healthy, and fat dog, three years old, was fed on white sugar, and distilled water, he was allowed to take as much as he could of each. For the first seven or eight days this diet appeared to agree perfectly well with him, but in the second week, although his appetite was always good, so that he eat six or eight ounces of sugar in the twenty-four hours, he grew thinner, his stools were neither frequent, nor copious, but his urine was secreted in abundance. In the third week the emaciation increased, he became weak, dull, and had less appetite; a small ulcer formed in the centre of each cornea, which penetrating them, evacuated the humours. He gradually became more emaciated and weaker, until he could neither eat nor swallow liquids, and died on the 32d day. The body was carefully examined, there was a total deficiency of fat, the muscles were reduced to five-sixths of their ordinary volume, and the stomach and intestines were very diminished in size, and strongly contracted. The gall-bladder and urinary bladder were distended with bile and urine which were sent to M. Chevreul to analyse; he found that they had all the characters of these fluids in herbiverous animals, that is, that the urine instead of being acid, as in the carniverous animals, was sensibly alkaline without a trace of uric acid, or of the phosphates; the bile contained a considerable quantity of picromel, which is the case with the bile of the cow, and of herbiverous animals in general. Similar experiments were tried with other dogs who were fed with oil, butter, gum, &c. and always with the same results, the nature of the excrementitious fluids being entirely changed became nearly like those of the herbiverous animals. M. Majendie deduces from these facts an important practical principle, that there is an evident relation between the diet and the presence of uric acid in the urine, or in other words, that no uric acid exists in the urine of those animals who do not eat flesh or other azotized food. M. Chossat, in a paper published in the fourth volume of the Journal de Physiologie, has confirmed this by numerous experiments on his own urine. Bérard, of Montpellier, has recently analyzed uric acid, and, according to him, 100 parts are composed of—

| | |
|---|---|
| Nitrogen | 39,16 |
| Carbon | 33,62 |
| Oxygen | 18,89 |
| Hydrogen | 8,34 |
| | 100,00 |

Pure uric acid is solid, of a pale yellow colour, heavier than water, without taste or smell; it is not decomposed by the action of air or water; boiling water only dissolves 1-1150th of its weight, and, consequently, the urine at 60°, which is its temperature in a state of health, will hold in solution only about 1-1500th of its weight of uric acid.

*To ascertain the Causes of Red Gravel,* the circumstances which determine the separation of uric acid from the urine in which, in health, it is in a state of solution, must be investigated. Three evident causes can diminish the power the urine possesses of dissolving uric acid; these may not be the only causes, but they are the chief. 1. Augmentation in the quantity of uric acid, the quantity of urine remaining the same, or not augmenting in proportion to the acid. 2. Diminution in the quantity of the urine, that of uric acid remaining the same, or not diminishing in the same proportion as the urine. 3. Diminution in the temperature of the urine, its quantity either remaining the same, or undergoing the changes above-mentioned.

1. *Causes tending to augment the Proportion of Uric Acid, and to produce in consequence Red Gravel.* Amongst the most evident is high living; thus, lovers of good eating, who indulge in large quantities of meat, fish, game, and highly azotized dishes, and who have passed the age for muscular exertion, are the frequent subjects of gravel. The following case illustrates this proposition. A merchant in one of the provincial towns of France possessed, in 1824, a considerable fortune; he was fond of the luxuries of the table, and was tormented with gravel and gout; owing to political misfortunes he lost his property, and fled into England, where he spent a year subject to extreme privations, but his gravel and gout disappeared; gradually his affairs became more prosperous, he resumed his good living, and with it the concomitant disease; by a second misfortune he lost all that he possessed, and returned to France without resources, his diet was as scanty as his means, and the gravel disappeared; by his industry he again raised himself to affluence, he returned to the pleasures of the table, and with them re-appeared the gravel and the gout, for which he consulted M. Majendie. Another proof of the influence of diet on the proportion of uric acid may be taken from individuals habitually moderate, who make an unusually large meal; the next morning, or even the same evening, their urine is strongly-coloured, and deposits a considerable quantity of uric acid. If sedentary and elderly men, who use but little their muscular system, take very substantial food, they are more likely to be affected with gravel than those who exercise their muscles vigorously, for the muscular system, when fully exercised, is that in which nutrition is most active, and which requires the most azotized nutriment; if the same diet is taken, and in considerable quantity, without exercise, the muscles do not require the azotized nutritive matter, it is in excess in the economy, and is directed to the principal emunctories of nitrogen, the kidneys, where it is converted into uric acid, and forms gravel. As long as the quantity of urine is sufficient to dissolve the uric acid, it produces no inconvenience, and this is the case with many persons who indulge in this luxurious diet; but if the proportion of urine diminishes, uric acid is immediately deposited.

2. *Causes which augment or diminish the Quantity of Urine, and which are favourable or unfavourable to the Deposition of Red Gravel.* Putting aside the chemical nature of the fluid, it is true that the more is drunk, the more abundant is the urine. This is correct as far as relates to those liquids which contain a large proportion of water, as beer, cider, or diluted wine; but it does not apply to strong wines or spirituous liquors, which contain much more alcohol than water, nor wholly to warm drinks, as tea, coffee, punch, &c. which produce cutaneous perspiration. If, then, a man who eats much animal food, drinks much water, or weak and effervescing wines, the quantity of his urine will be more than sufficient to dissolve the uric acid formed by the kidneys, and he will be less exposed to be attacked by gravel. If, on the other hand, he drinks but little, or not in proportion to the food he takes, or if he drinks freely of heating wines, brandy, strong liqueurs, or any fluids highly charged with alcohol, his urine will be less abundant, and, consequently, will dissolve less uric acid; his urine will, therefore, be so much the more disposed to deposit gravel. If it was merely sufficient to drink much to avoid the gravel, those who are most subject to it (les grands mangeurs et les gastronomes) would suffer from it rarely. But a particular and imperfectly known cause acts upon them in an inverse ratio; this is, the diminution of the action of the kidneys by the use of animal food. In experiments which M. Majendie has made on animals to ascertain the effects of azotized and non-azotized food, he has found that the use of the latter augments sensibly the quantity of urine; thus, a dog fed with sugar, and a given quantity of water, makes, in 24 hours, a much greater quantity of urine than another dog, drinking the same proportion of water, and fed with meat. M. Majendie has often observed the same thing in man. Individuals who feed on meat alone, for the cure of any disease, are remarkable for the small quantity of urine they secrete. The contrary is the case with persons who are on a strictly vegetable diet. M. Clouet, wishing to make an estimate of the nutritive properties of the potatoe, eat that root alone for some time; at the end of 12 or 15 days he was seized with a diuresis, which had some analogy with diabetes. A further proof is deduced from the comparison of the quantity of urine secreted by carnivorous and by herbivorous animals, in the former it is much more considerable—thus, contrast a cat and a rabbit of the same size, although the latter never drinks, it secretes urine much more abundantly than the former. The fact which M. Majendie has endeavoured to establish, that meat and other analogous foods tend to diminish the quantity of urine, at the same time that they augment the quantity of uric acid, will be found to form the basis of his treatment. It is almost unnecessary to add, that all causes diminishing the quantity of urine, such as abundant perspiration, sweats, liquid evacuations, &c. will be favourable to the formation of gravel, by preventing the solution of uric acid. In the same manner, prolonged confinement to bed, either by exciting cutaneous perspiration, or by retarding the passage of the urine into the bladder, favours the formation of gravel. Van Swieten relates the case of a man who had never had any symptom of gravel, but was attacked with a calculous nephritic colic a few weeks after the cure of a fracture in the thigh, for which he had remained in bed and in one position for ten weeks. After great pain he expelled a small rough calculus, and became subject to gravel. Similar effects may be caused by habitually retaining the urine a long time in the bladder.

3. *Influence of the Temperature of the Urine on the Deposition of Red Gravel.* The tendency which old people especially have to deposit gravel is owing to the diminution of animal heat. M. Majendie has found, from thermometrical experiments, that after 60 years of age, the temperature of the body is reduced many degrees; it rarely exceeds 96° (Fahr.) even in the arm-pits or groin, which are the hottest parts of the surface of the body, and which follow almost always the temperature of the internal cavities. The urine is proportionally cool, Majendie has rarely found it above 86°, that is 14° or 18° below its ordinary temperature. From this circumstance alone, cæteris paribus, the old man is more exposed to gravel, as his urine is less able to dissolve uric acid. Perhaps the long continued action of cold in the adult may produce a similar effect.

2. *White Gravel.* This is found next in frequency to the red. It is deposited in 2 forms. 1. As a white powder. 2. As small angular and irregular gravel, of various consistence. This is almost entirely composed of phosphate of lime, with a little phosphate of magnesia. It dissolves readily in weak muriatic acid, and the phosphate of lime is precipitated without effervescence by ammonia. Prout describes white and friable concretions, formed almost wholly of carbonate of lime; these of course would be distinguished by their effervescing with acids.

Majendie has never found them in the urine of man, but in that of animals, particularly of the horse, they are common. If the experiment on the dog, fed on non-azotized substances, be referred to, it will be found that the presence of phosphate of lime in healthy urine is evidently connected with animal diet, for on depriving the animal of it for 20 or 25 days, no traces of the phosphates could be discovered in his urine. In fact, all the remarks of the causes which favour the deposition of the red gravel, are applicable to the white, formed of phosphate of lime. The presence of carbonate of lime, a principle which does not exist in healthy human urine, seems to depend on an exclusively vegetable diet, as this salt is found in the urine of herbivorous animals.

3. *Hairy Gravel.* This exists either in the form of a whitish powder, mixed with hairs, or as gravel of various sizes, hairy on the surface, and sometimes connected together as a bunch of grapes. In the powdery state, the small hairs which are mixed with the white powder vary in length, from a line to an inch, or more; they differ little from ordinary hairs, being only a little finer, and of a cindery-grey colour. The saline matter forms a layer at the bottom of the vessels; if it is left for some time it cakes, and can only be detached in layers of some lines in thickness; on breaking these, the hairs are seen on the edges of the fracture. M. Pelletier analysed this, and found it to consist of phosphate of lime, with a little phosphate of magnesia and traces of uric acid. The first case in which M. Majendie observed this peculiar disease was of a professor of the university; the deposition was so great that he filled, in some days, a vessel which held more than a pint; the quantity which this man had passed in several years was truly extraordinary. The second case was that of a sailor, who evacuated gravel connected by hairs. In neither of these cases, nor in a third, which will be detailed when the treatment is discussed, could excess in animal food account for the production of the phoshates; they were very sober men. M. Majendie offers no explanation of the source from whence these hairs were derived. He does not appear to have been aware that Hippocrates had observed a similar deposition in the urine, *οκοσοισι δε εν τω ουρηματι παχει εοντι, σαρκια μικρα ιριχοειδεα συνερχεται, ταυτα δε απο των νεφρων ειδεναι χρη εοντα και των αρθριτικων.—(Hipp. Sec.* 3, *p.* 230. *Tom.* I. *folio. Geneva,* 1657.) Hippocrates does not assert that they are actual hairs, but that they are small fleshy bodies like hairs, in thick urine—that they come from the kidneys, and from those persons affected with gouty disease of the joints.

4. *Grey Gravel.* Majendie has never seen this deposited as sand; in some cases, the concretions were smooth and similar in form, and nearly the size of an olive or pistachio nut, in others spherical and rough; they are formed of concentric layers, indicating the slowness of the deposition; they are true urinary calculi, but their size is not voluminous enough to prevent their expulsion through the urethra. They are composed of the ammoniaco-magnesian phosphate, united to a small quantity of animal matter, and some traces of uric acid. From the presence of ammonia, it is probable that a highly azotized diet is the chief source of this gravel; M. Majendie has never observed it, except among "veritables gastronomes."

4. *Yellow Gravel.* This, which is composed of oxalate of lime, is rare. M. Majendie has seen but one instance of it; the gravel stone which was expelled was about six or seven lines in length, and from 1½ to 2 lines in breadth, elongated, flattened on two sides, hard, of an orange-yellow colour, and uneven surface; M. Desprex, an excellent chemist who examined it, found that it consisted of oxalate of lime, nearly pure. M. Majendie was surprised to find such a product in an individual whose corpulence, age, habits of life, &c. were favourable conditions for the secretion of *uric acid*, and, therefore carefully investigated his diet. His patient, who had always indulged in the pleasures of the table, had been on a political mission to a neighbouring country, where "le goût de la bonne chère est fort répandu," and had received and given numerous official dinners, where he had shewn himself "à la fois diplomate habile et gastronome éclairé." On his return he gave up his official station, and on entering into private life, he resolved to reform his habits and to adopt a cooling regimen. For this purpose he took every morning a large plate of sorrel, and continued this nearly a twelvemonth; at the end of this period he was attacked with a fit of gravel, and expelled the oxalate of lime calculus. Its origin was thus readily traced to the immoderate use of sorrel.

6. *Transparent Gravel.* These concretions are composed of cystic oxyde, and are very rare. They are of a yellow, citrine colour, and of the transparency of topaz; on breaking them they appear to be composed of an aggregation of crystals, mingled together without order. On burning, they exhale a fetid odour; they are soluble in acids and alkalies. Cystic oxide was discovered by Wollaston. M. Lassaigne analysed a specimen found in a dog's bladder, and the following was its composition:

| | |
|---|---|
| Carbon. . . . . . . . . . . . . . . . | 36.2 |
| Azote. . . . . . . . . . . . . . . . . | 34.0 |
| Oxygen . . . . . . . . . . . . . . . . | 17.0 |
| Hydrogen . . . . . . . . . . . . . . | 12.8 |
| | 100.0 |

The chemical nature of this gravel connects it evidently with uric acid, both as to its origin and relation with animal nutriment.

*Of some of the particular Causes of Gravel.* M. Majendie allows that there are many causes of gravel which he cannot explain by the physiological or chemical laws; he admits that we see individuals daily, who, from their age, diet, and habits, present the most favourable conditions for the formation of these concretions, but who are not subject to them; and that, on the other hand, there are examples of persons attacked with gravel, whose diet and modes of life are very different from those who are generally the subjects of it. Thus, according to Scudamore, gravel is frequent among the poor between Tunbridge Wells and Lewes in Sussex, although their food is almost exclusively vegetable, with hard beer, and yet it spares the better fed inhabitants. Gravel is not unfrequently expelled after making a violent exertion, also, by individuals sober and otherwise healthy, if they have indigestion, accompanied by bitter or acid eructations, pyrosis, &c. M. Majendie met with an instance of a boy, who expels two drachms of red gravel the day after he has eaten a salad, and Béclard informed him of an individual who discharged one or two small calculi invariably after eating unripe fruit. He believes that dyspepsia and gravel, which exist simultaneously in the same subject, are effects of the same cause, and do not reciprocally produce each other. It has been for a long time observed, that the inhabitants of temperate and of moist climates were most subject to calculous affections, and that in cold and equatorial countries it is rare, and authors have attributed this to the difference in temperature. M. Majendie has been led to believe that this absence of calculous disease depends on the vegetable diet, from the following fact, related to him by M. Orfila. The inhabitants of the Island of Minorca live principally on fish and other animal food, they season these substances highly with pepper and other spices; their drink is a generous wine, which they rarely dilute, they also use freely brandy, rum, and punch. During Orfila's residence there, in 1816, he remarked that calculous diseases were very common, and that the urine of the inhabitants was highly charged with uric acid, which produced small calculi, that were expelled with the usual symptoms. Gravel has been believed to have been caused by the stony concretions of some fruits, particularly of pears, but Maquart and Vauquelin have studied their chemical composition, and find that they are neither composed of phosphate nor of carbonate of lime, nor of uric acid, as had been suspected, but of a woody matter like that of the tree which produced the fruit, confusedly crystallized, and mixed with a kind of starch. Thus they could not be a cause; and, besides, Majendie has frequently found that they have passed through the intestines without undergoing any change. Hales has stated that gravel often was produced by drinking waters containing carbonate of lime; but it appears from the reports of Chopart and Dessault, who practised surgery in the largest hospitals of Paris, that stone or gravel was very rare in the village of Arcueil, although its water is impregnated with carbonate of lime. Besides, the composition of calculi is not similar to this salt. The same argument applies to common salt, which many authors have stated to be one cause. To recapitulate,—the causes of gravel, either direct or indirect, are, 1. Mature or old age. 2. Too nutritious diet, principally composed of substances containing much nitrogen, or of substances capable of forming deposits in the urinary passages. 3. Want of exercise, literary studies, confinement to bed, &c. 4. A small quantity of drink. 5. Generous wines and strong liquors. 6. Perspiration, abundant sweats, and all serous evacuations in those disposed to gravel. 7. The habit of keeping the urine a long time in the bladder. 8. Some particular causes, whose effects are obvious, but whose modus operandi is unknown.

*Symptoms of Gravel.* Most frequently, some months before the expulsion of gravel, a sense of numbness and uneasiness is felt in the region of the kidneys, the urine is deeper in colour, and deposits a reddish sediment on cooling. These symptoms attract but little attention, but they increase; there is greater pain and weakness in the loins, and the day after this pain has been most severe, a certain quantity of sand is evacuated with the urine; this is frequently attended with heat and burning pain in the course of the urinary passages, sometimes the pain is acute and is attended with fever. The symptoms generally become more severe, the pains in the kidneys increase in violence at each attack, and at times are intolerable, the patient tracing the torturing descent of the stone through the ureter; this is almost always accompanied with frequent desire to make water, retraction of the testicle, cramps, nausea, and vomiting; the patient cannot remain in the same posture, he cannot stand or walk: this remains 36 or 48 hours, and suddenly ceases. During the day the patient expels, with some difficulty, one or more calculi. From these general symptoms the important deduction may be drawn, that the solidification of gravel takes place as soon as the urine is formed, that is, in the pelvis of the kidneys, and perhaps, as the numbness and uneasiness in the loins seem to indicate, in the tubular structure of the kidneys themselves. M. Majendie found, in the tubular structure of the kidneys of an old woman, who was subject during her life to gravel, small concretions of uric acid, varying in size, from fine sand to elongated calculi, an inch in length. Chopart (Maladies des Voies Urinaires) says that he has found gravel stones in the tubular structure of the kidneys. It is probable that the solidification, in some instances, takes place either in the ureter or bladder. If, from its size or shape, a portion of gravel is arrested in its descent, it impedes more or less other particles; this is generally the origin of renal and vesical calculi. Stones in the kidneys, ureters, and bladder are most frequently the sequelæ of gravel, or its third degree, the expulsion of sand being the first, and of gravel the second.

*Treatment of Red Gravel.* There are four principal indications.—1. To diminish the quantity of uric acid which the kidneys form. 2. To augment the secretion of urine. 3. To prevent the solidification of uric acid by saturating it. 4. The gravel being formed, to favour its evacuation and to attempt its solution.

1. *To diminish the quantity of Uric Acid.* As the existence of this acid in the urine is connected with the use of food containing azote, it is necessary to diminish the quantity of the latter in order to prevent the undue secretion of the former. Majendie has frequently seen those cases in which the urine is loaded with red sand, cured by the patient's leaving off meat breakfasts, that is, if they did not make up at dinner for the morning's privation. At the commencement of this restriction the patient often feels weakness and malaise, but after a few days he finds the benefit, both in the increase of his spirits, and of general comfort. Those persons who make but one solid meal should diminish that one quarter or a half; this simple and efficacious remedy is difficult to put in practice for many persons, and particularly for old men; the dinner is the most important act of the day, a period of true and positive enjoyment. This diminution may be supplied by less hurtful food, such as bread, pastry, farinaceous articles of diet, rice, potatoes, green vegetables, sugar, &c. but still the patient must not abuse the use of these, as some bread and pastry contain much azote. Strong liquors and pure wine must be avoided, and aqueous drinks plentifully taken. Too often, notwithstanding these precautions, the gravel persists, and is very painful, and the stones are large and numerous; the patient must then be wholly deprived of azotized food. To give a general idea of the diet he recommends, M. Majendie has given an extract from the letter of a gentleman who was addicted to the pleasures of the table, and suffered severely from gravel, for which Majendie recommended a non-azotized diet. "On rising I take a cup of weak tea, in the course of the morning some 'orgeat,' (a drink whose chief ingredient is almonds); I dine at noon off vegetables and fruits, drinking many glasses of water with old white Bordeaux wine, and at dessert, two or three small glasses of the same wine undiluted; at five o'clock I drink sugared water; in the evening I take rice, or 'bouillie de blé noir,' and sometimes gruel, made with water and butter; I drink with this meal some diluted white wine, and before going to bed a glass or two of sugared water." At the end of six weeks the gravel disappeared. After continuing this diet for 12 or 15 days the patient made an abundant quantity of urine, more than in proportion to the drink. A similar phenomenon was observed in the experiments on the dogs.

2. *To Augment the Secretion of Urine.* The most simple method is to drink abundantly of those fluids whose base is water, and particularly those known to be diuretic. Various vegetable decoctions, mineral waters, &c. have been recommended as specifics, but that must be chosen which proves diuretic, and agrees with the patient's digestion, and suits his taste. Five or six pints of such a liquid taken daily is not too much, especially if the gravel is severe. This sometimes produces a loss of appetite, laborious digestion, and general debility; the quantity should then be diminished, and aromatic or iced drinks substituted.

3. *To Saturate the Uric Acid.* When non-azotized diet, and abundant drinks have failed, recourse must be had to remedies which act by combining the uric acid with an alkaline or earthy base in order to form salts much more soluble than the lithic acid itself. It is well known that certain substances taken into the stomach impart very quickly to the urine their peculiar qualities, either of smell, as asparagus; of colour, as rhubarb; or are found in the urine without undergoing any alteration, as certain saline substances. Darwin found nitrate of potash in the urine half an hour after a few grains had been taken; and Majendie has confirmed these experiments both in man and animals. In order to saturate uric acid, alkaline carbonates are to be employed, whose bases are in excess, the uric acid combines with the excess of base, and urates are formed with the greatest facility, as a small quantity only of the base is necessary to saturate the acid. Even when the uric acid is saturated all fears for the deposition of sand are not removed, for the urates are only soluble in an excess of their bases, and are decomposed by the weakest acids; it is necessary then to have an excess of alkali in the urine in order to prevent the formation of a new species of gravel by the deposition of the urates. This important result is promptly and easily produced in carnivorous animals, whose urine, like that of man, is acid. Majendie has many times found it alkaline in dogs two hours after they had swallowed a certain quantity of carbonate of lime, soda, or potash, and the same phenomenon is not more difficult to produce in man ; pure alkalies have a similar action, but they cause greater irritation of the bladder. The carbonates of soda and of potash being soluble in water, in all proportions, may be given dissolved in a large quantity of fluid, in a saturated solution, or in the solid form ; but as carbonate of lime and magnesia are not soluble, they must be taken in the form of a powder, or suspended in water by means of mucilage ; their insolubility renders them less efficacious than the former, and sometimes they are not absorbed but form concretions in the alimentary canal. The dose of carbonate of lime and of magnesia may be carried to many drachms in the 24 hours, some have taken an ounce. If more than from 24 to 36 grains of carbonate of soda or potash are taken daily, the stomach is apt to become deranged, and vomiting to come on, which is seldom the case if the dose is less considerable. Soda or potash, from their causticity, should be prescribed with greater precautions ; they must be diluted so that the solution may make but a slight impression on the tongue, and a pint of this liquid may be taken daily for the same purpose. Magnesia may be given in the dose of 10 grains to an ounce or more in the 24 hours, either suspended in water or in boluses. Mineral waters containing earthy or alkaline carbonates act usefully in gravel by exciting the secretion of urine, for the small quantity of alkali they contain renders it improbable that it can saturate the uric acid.

This observation does not apply to the waters of Vichy, which contain a large proportion of bicarbonate of soda, which speedily renders the urine alkaline, as appears from the experiments of M. Darcet, in a paper published in les Annales de Chimie, Mars, 1826. M. Majendie has several times observed that an alkaline bath has rendered the urine alkaline. Experiment will alone determine which alkali will agree best with the patient, and will most effectually answer the desired end. The employment of alkalies in the cure of gravel is attended by the most marked and prompt success, but unless the diet is changed and the causes of the disease removed, it is a palliative merely, whose effects after a certain time will completely cease. In a physiological point of view the action of the alkalies, taken into the stomach, on the urine deserves attention. The surface of the stomach and intestinal canal being supplied with an acid which is constantly renewed, how is it that the alkalies do not combine with this acid, and that the carbonates are not decomposed? These combinations do take place, but the quantity of acid in the stomach and intestines is so great, and absorption so rapid, that the greater part of the alkali is absorbed and taken into the blood before it is acted upon by the acid of the digestive organs. Once mixed with the blood it can undergo no decomposition, as the blood itself is alkaline. But it is not so easy to explain the passage of an acid from the stomach to the bladder, for as soon as it arrives in the veins and mixes with the blood, it must necessarily combine with the excess of alkali in the serum. Is not the fact of the passage of acid from the intestinal canal into the urine still doubtful? M. Majendie does not regard this as impossible, but he thinks that experiments have not proved its truth. Although carbonic acid has been procured from the urine of a man who had previously drunk water saturated with this acid, it does not necessarily follow, that it had passed from the stomach to the kidney, as urine contains, when in a healthy state, carbonic acid. M. Magendie has frequently tried to neutralize alkaline urine by giving mineral or vegetable acids in strong doses, but he has never been able to obtain the result in a non-equivocal manner.

4. *To favour the Expulsion of Sand and Gravel, and to attempt their Solution.* As soon as the pain and uneasiness in the lumbar region, or the expulsion of a small quantity of sand, announces the existence of urinary concretions in the kidneys, means must be used for their expulsion. The small size of the grains of red sand renders their expulsion less difficult than the other varieties; in most cases it is sufficient to drink freely of water, or some other aqueous drink to increase the quantity of urine; some take for this purpose a large glass of Seltz water, or of weak beer, or very diluted wine, either at night or in the morning; and with this simple precaution are able without inconvenience to enjoy the pleasures of the table, and to expose themselves with impunity to the causes which produce gravel; but the majority are not so fortunate, and are obliged to follow the necessary means for the diminution of uric acid, otherwise they are harassed by constant pains in the kidneys, producing want of sleep, and general disturbance; momentary relief may be obtained by leeches or venesection, but permanent benefit cannot be expected unless the diet is changed. Some patients consider that the formation of sand ensures them against renal and vesical calculi, and in consequence neglect these rules of diet, but this opinion is without foundation; it is not rare to find calculi in the bladder of those who have discharged sand for many years, and there are authentic cases of the ureters having been obstructed by the accumulation of very fine gravel. Calculi, often of a considerable size, have been discharged periodically during many years with no more inconvenience than a slight impediment to the flow of urine at the moment of the exit of the calculus; probably the ureters of such patients are large, and the concretions smooth; even in these cases it is advisable to drink every day a certain quantity of aqueous drink to render the urine more abundant, and to prevent the possibility of the calculus being impeded. Such patients will derive advantage from exercise, either on foot or horseback, and in rough vehicles; of course such means would be injurious if the passage of the calculus is attended with pain. For the same purpose an emetic may be given in order to produce strong contractions of the abdominal muscles. Usually the passage of a gravel stone from the kidneys is attended with the acutely painful symptoms, and when this is the case, the expulsion of the calculus must not only be favoured by drinks, but the irritation should be subdued by complete abstinence, general bleeding, leeches, cupping, local and general baths, emollient fomentations, &c. according to the violence of the symptoms, and the age, temperament, and strength of the patient. Generally after 36 or 48 hours the symptoms abate, and one or several calculi are expelled; in some cases the attack is prolonged 10 or 15 days. If the remission of pain, &c. is not followed by the expulsion of a calculus, it is probable that the unfavourable symptoms will recur. Every remedy should then be employed for the evacuation of the retained calculus, as diuretic drinks, baths, dry frictions over the loins and in the direction of the ureters, exercise on foot, or on horseback, or in a carriage, if the patient can bear it. Emetics sometimes succeed when other remedies have failed. If the situation of the pain announces that the calculus has traversed the ureter and is arrested in its inferior extremity, a metallic sound may be introduced into the bladder, to endeavour to detach it, or the finger into the rectum to attempt to displace it by striking the back of the bladder. If the calculus is detained in the urethra, judicious pressure along the canal, oily or emollient injections into the passage, and abundant drinks, which often are alone sufficient to expel it; in some cases surgical aid is requisite. Notwithstanding these means the calculus may remain in the pelvis of the kidney, or in the bladder; its increase must then be prevented by putting the patient on a non-azotized diet, and the urine must be maintained alkaline, to endeavour to produce a solution of the concretion. Supposing the calculus to consist of uric acid, both physiology and chemistry lead us to suppose that these means will succeed. Experiments have shewn that a mass of uric acid plunged in urine, which is saturated with potash or soda, with an excess of alkali, and kept at a temperature of 77° to 95° Fahrn. which is frequently renewed, is in time completely dissolved. There is no positive proof of the solution of a renal calculus, but the many cases of calculous nephritis, which have been cured by the use of alkaline remedies, render it probable. This only applies to those formed of uric acid. M. Robiquet, an expert chemist, has published a case in which it was probable that a red calculus was dissolved by alkalies.

J. B. M., æt. 74, a retired merchant, had all the symptoms of stone in the bladder; Marjolin sounded him, and found a stone which was small and soft; he recommended him to have it extracted by Civiale's method; but M. Robiquet and Dr. Farrot, whose patient he was, put him under the following plan of treatment:—to drink daily two pints of a solution of bicarbonate of soda, (5 grammes, ʒj. grs. xvij. to the pint) and to take frequently hip-baths, and emollient glysters. He was recommended to abstain from excitants. After a few days he was better, his urine was more abundant, its emission was rarely preceded by pain, and there was less irritation of the bladder. In 15 days the baths and lavements were discontinued; in a month the patient considered himself so well that he could be with difficulty persuaded to continue to take a pint of the solution per diem. Three months after the commencement of the treatment he felt acute pain in the urethra and expelled a small calculus, the form and size of a lentil; this was wholly composed of lithic acid, having all the appearance of the nucleus of a larger calculus, which had been gradually worn away. Marjolin, as the patient felt no pain or inconvenience, declined sounding him again.

*Treatment of White Gravel.* There are two varieties, one consisting of phosphate of lime, the other of carbonate of lime. 1. Gravel composed of phosphate of lime may be successfully treated by diet and drinks. The diet is the same which has been recommended in red gravel, for it has been shewn, that the urine of carnivorous animals who have been kept on non-azotized food, loses all traces of its phosphates. The drinks should consist of fluids charged with carbonic acid, which taken in great abundance augment the quantity of urine, and tend to dissolve the phosphate. For this purpose, either artificial or natural Seltz waters, or the waters of Contrexeville, Bains, or even Vichy, may be recommended. By such treatment Majendie has known this white gravel disappear in a few weeks, he has had no personal experience as to the efficacy of the diluted mineral acids, and he has known many affected with gravel who have derived no advantage from their use. M. Majendie has never seen white gravel composed of carbonate of lime; its treatment will consist in discovering the cause of the deposit, and in administering drinks strongly impregnated with carbonic acid, in order to dissolve the carbonates which are soluble in an excess of that acid.

*Treatment of Hairy Gravel.* M. Majendie has seen three cases; two of these yielded readily to a vegetable diet, and the carbonated alkalies; the third was more obstinate. This patient applied to M. Majendie in 1827. He had suffered from gravel for ten years, and after describing the acute pain which attended micturition, he writes, "but that which strikes me as a new feature in my case, is, that my urine, which is clear enough when I evacuate it, is no sooner cooled than it deposits a prodigious quantity of calcareous matter, which when dried appears like a thick layer of a friable grey sediment, the different particles of which, when attempted to be separated from each other, are held together by an infinite number of small hairs, something like hemp." On analyzing the sediment it was found to be composed of phosphate of lime united to a small quantity of phosphate of magnesia. M. Majendie recommended a slightly azotized diet, with carbonate of soda, and the patient's condition was much improved. The hairs first ceased to be observed in the deposit, and the quantity of the phosphates gradually diminished. Owing to a stricture of the urethra the bladder was too irritable to bear the alkalies for a length of time sufficient to ensure his cure.

*Treatment of Grey Gravel, composed of the Ammoniaco-Magnesian Phosphate.* It is not very rare, but it is one of the most painful and dangerous forms, as the particles of gravel are often large and rough. It may be cured by regimen alone: by putting the patient, who is generally a lover of the table, on a moderate diet, he will be much relieved, and, if this does not succeed, the strictly vegetable diet, as recommended in red gravel, will certainly produce a complete cure, if the individual has sufficient resolution to submit to the restriction.

*Treatment of Yellow Gravel (Oxalate of Lime).* M. Majendie has only met with the case which has been detailed, and which was traced to the eating of sorrel; interdicting the use of this vegetable produced a complete cure. Professor Langier communicated a somewhat analogous case to the "Academie Royale de Médecine" some years since. The father of a celebrated artist in Paris, who had been sounded, and a stone detected in the bladder, called upon him to know if he had no chemical agent which would supersede the necessity of an operation; he recommended him to have the operation performed, and it was successful. After some months the patient returned, and requested to know whether some remedy might not be recommended to prevent the return of the complaint; the patient shewed the stone, which consisted of oxalate of lime. M. L. questioned him as to his diet, and found that he was very fond of sorrel, and that he eat it daily; he was of course recommended to leave off this article of diet, as it contained an acid of which the calculus was partly composed.

*Treatment of Transparent Gravel (Cystic Oxyde).* M. Majendie has seen but one case; this was much benefited by a vegetable diet and alkalies; this treatment was founded on the consideration of the chemical nature of cystic oxide, which, according to Lassaigne's analysis, contains, in 100 parts, 34 of azote. M. Majendie considered that, by diminishing the quantity of azote in the food, he should prevent the excessive formation of a principle which forms one-third of the diseased deposition; and as cystic oxide is soluble both in alkalies and acids, he attempted, by rendering the urine alkaline, to dissolve the foreign body if it existed in a solid state.

*Mixed Gravels and their Treatment.* Proust mentions the case of a man who expelled gravel stones of phosphate, and others of carbonate of lime. M. Majendie attended a patient, who was affected with sand consisting of phosphate of lime and magnesia, who stated that he had been previously subject to red gravel. Alternating calculi prove the same thing. This is probably a less rare affection than is supposed; but physicians often neglect the analysis of gravel. M. Majendie, towards the conclusion of this volume, pays a verbal compliment to the work of Dr. Prout, but he does not appear to have paid him the compliment of reading his book, otherwise he would hardly have stated his *one* case of mixed gravel, and his *supposition* that it is not so rare as is supposed. Dr. Prout most distinctly states, that the deposition of the phosphates is rarely original, but that it is a state induced by, or consequent to, the other forms of urinary deposition, especially the lithic acid and oxalate of lime; he also describes the symptoms which mark the transition from the lithic to the phosphatic diathesis! M. Majendie does not vary his treatment in these cases.

*Prostatic Calculi.* It is of importance to distinguish these from gravel. They are never larger than a small pea, having almost always distinct and regular facets, which are produced by the attrition of many in one cell; at other times they are fusiform, and then there is but one calculus in each cell.

*Vesical Calculi* The largest vesical calculi are only very large gravel stones; they have commenced by small calculi, which, from some mechanical impediment, have been detained in the bladder, and have increased by the addition of successive layers; even when these are removed by lithotomy or lithotrity, the immediate cause of pain is got rid of, but the disease is not cured, nor even attempted to be cured; consequently, calculi very frequently form again, and require another painful operation. Surgeons have not sufficiently attended to this point; as soon as the stone is extracted it should be submitted to a careful analysis, and a treatment adopted according to the nature of the gravel which formed its nucleus. Prout has proved, that two-thirds of the total number of calculi are formed of uric acid, or have a uric acid nucleus; therefore, in the majority of cases, the treatment for red gravel is to be recommended: if the calculus is found to consist of any of the other principles, the patient will require the same remedies which were recommended for similar sorts of gravel.

M. Majendie devotes a short chapter to the "empirical treatment of gravel;" he confesses that, as no theory will comprehend the explanation of all the causes of calculus, or the mode of action of all the curative remedies, the most experienced physician must have recourse to means of cure, whose efficacy has been proved by experience, although it cannot be accounted for by reasoning. Thus, he says, these patients are benefited by remedies which are useful in relieving the dyspepsia which often accompanies their disease, such as small doses of rhubarb and magnesia, quinine, repeated purgatives, &c. Other causes, such as change of air and occupation, relief from mental anxiety, &c. act beneficially, but no plausible explanation can be given of their mode of acting.

We have now fulfilled our promise, and have given the whole of the practical information contained in M. Majendie's work. It is valuable, from its accounting, on physiological principles, for the action of animal food in the production of uric acid, and it contains many judicious hints, which may be made available in practice, but M. Majendie simplifies too much, he endeavours to trace the origin of every species of gravel to the same cause, and to enforce a similar treatment; his mechanical notions of imbibition have influenced him too strongly, and he looks on the human frame rather as a piece of human mechanism, than as a body endowed with life. He founds his practical observations on comparative physiology, but even here there are startling exceptions, the urine of the lion and tiger, although they are carnivorous animals, contains no uric acid, whilst that of the boa constrictor is almost wholly composed of it. Foucroy and subsequent chemists have also found uric acid in the urine of domestic fowls. There are similar exceptions in practice; although the bon vivant may be benefited by complete abstinence from animal food, yet the debilitated sufferer from depositions of the phosphates, according to Dr. Prout, a high practical authority on this point, is relieved by a moderate animal diet more than by an acescent vegetable one. The same obscurity hangs over the action of some medicines; Dr. Prout forbids the use of saline purgatives, containing a vegetable *acid*, in the phosphatic diathesis, as he has observed that they render the urine *alkaline*. Surely such instances prove that the vital action of the digestive or urinary organs of different animals, in a physiological state, and of different individuals in a pathological condition, produce some change in the ingesta more important than the mere mechanical separation of their elements, which M. Majendie alone recognizes? Besides, there are other sources by which nitrogen is conveyed into the circulating fluid, and which M. Majendie has not mentioned—respiration and absorption. The experiments of Dr. Edwards on this subject, proving that the absorption and exhalation of this gas by the lungs varies in different temperatures, ages, seasons of the year, &c. may perhaps cast some light on these diseases, and explain more satisfactorily some of their causes. Although M. Majendie allows that there are many remedies which have a beneficial effect on gravel, whose action he cannot explain, yet he does not appear to have tried them in his own practice, and is contented with the mere enumeration of them. We suspect that the practical opportunities which he has had of testing the truth of his physiological speculations have been confined to one class of individuals, those who are addicted to the pleasures of the table, otherwise he would hardly have asserted that a restricted diet was a certain cure. Notwithstanding this Sangrado simplicity of treatment, so exclusively recommended in all cases, and which forms a strong contrast to the manner in which Dr. Prout discusses the same subject, there are many obscure causes of gravel mentioned by him, for which M. Majendie satisfactorily accounts, such as the deposition of a red sediment after hard exercise, an unusually large meal, &c. which all who have a susceptibility to these depositions must have constantly observed; the greater frequency of gravel in old people, &c. So little is at present accurately known of the manner in which medicines or food act on the various organs of the body, that any successful explanation of their modus operandi must be hailed with pleasure; this is one of the great practical benefits to be expected from the study of physiology, and we are happy to recommend to our readers a useful and instructive work, in which this application has been made by one of the most celebrated experimental physiologists of France.

